# Ergonomics Risk Assessment of Musculoskeletal Disorder During Ultrasound-Guided Internal Jugular Venous Cannulation

**DOI:** 10.21315/mjms2024.31.5.13

**Published:** 2024-10-08

**Authors:** Abdul Hafiz Dzulkafli, Shaik Farid Abdull Wahab, Rohayu othman

**Affiliations:** 1 Department of Emergency Medicine, School of Medical Sciences, Universiti Sains Malaysia, Kelantan, Malaysia; 2 Hospital Universiti Sains Malaysia, Universiti Sains Malaysia, Kelantan, Malaysia; 3 MARA High Skills College, Kelantan, Malaysia

**Keywords:** ultrasound, cannulation, ergonomics, musculoskeletal disorder, REBA

## Abstract

**Background:**

Acutely sick patients can receive emergency intravenous access through central venous cannulation to administer fluids and medicines, perform haemodynamic monitoring and extracorporeal therapies, including plasmapheresis or haemodialysis. Using the Seldinger procedure, access is gained by percutaneous puncture, frequently guided by ultrasonography into the femoral, subclavian or internal jugular veins. This study aimed to identify ergonomic risk factors for musculoskeletal disorders (MSDs) in operators performing ultrasonography-guided internal jugular vein (IJV) cannulation at various table heights and probe orientations.

**Methods:**

Sixty emergency medicine residents participated in a cross-sectional study conducted by the Emergency and Trauma Department of Hospital Universiti Sains Malaysia, Kelantan. Participants were instructed to perform the cannulation at two distinct table heights and with two distinct probe orientations. To compute the ergonomic risk score, the Rapid Entire Body Assessment (REBA) method was used.

**Results:**

The table height of 0.5 elbow factor with varied probe resulted in a median REBA score of 5.0, whereas the table height of 0.7 elbow factor with varied probe had a median REBA score of 4.0. All four positions exhibited medium risk for MSDs.

**Conclusion:**

This study showed that the table height of 0.7 elbow factor is more ergonomically favourable while still imposed medium risk for MSDs.

## Introduction

An intravenous cannula, at the emergency department (ED), is frequently inserted centrally or peripherally. Despite being quicker and easier, peripheral access may not frequently be favoured over central venous cannulation (CVC) (1). CVC is typically used for emergency venous access in cases of problematic peripheral venous access, for infusion of large volumes of fluids or irritating medications, for close haemodynamic monitoring of central venous pressure or cardiac output and as an intravascular access for haemodialysis (2, 3).

The three main locations for CVC are the internal jugular (IJV), subclavian and femoral veins. Each site has benefits and drawbacks. Internal jugular CVC is typically favoured over femoral CVC, which has a higher risk of infection and subclavian CVC, which carries a risk of bleeding and pneumothorax. The use of ultrasonography as guidance has increased the cannnulation success rate and reduced the risk of complications (3).

Several studies have shown that ultrasonography-guided CVC reduces complications and improves the safety and quality of CVC implantation (4); however, research on operator ergonomics is limited. Ergonomics is the scientific study of how individuals physically interact with their work environment, which includes the design of equipment and the training of individuals in terms of their motor, visual, spatial and hearing capacities (5).

In emergency situations, ultrasonography is used for diagnosis and treatment. Regular imaging guidance in minimally invasive procedures such as CVC may cause ergonomic strains and stress injuries. An incorrectly designed workstation may cause terrible posture and musculoskeletal concerns for the operator. Soares et al. (6) suggested that an ergonomically unsuitable workplace can cause acute and chronic pain, mental strain, poor performance and low-quality work.

Considering the increasing number of medical professionals reporting MSD disorders such as wrist, neck and back pain, it is important to determine the manageable and treatable causes of musculoskeletal disorder (MSD) experiences inside the department. Therefore, operator fatigue and pain can be avoided and eliminated with early intervention, thereby improving performance and workplace positivity.

Medical workers are prone to work-related musculoskeletal disorders (WMSDs) (7). Approximately 33% of medical personnel’s sick leave is because of MSDs (7). Emergency doctors and paramedics are at a risk of MSDs owing to their work environment (8). Previous studies linked WMSDs to repetitive stress injuries from cardiopulmonary resuscitation (CPR) and improper posture during intubation (8, 9).

Baker and Coffin (10) observed the incidence of WMSDs had gradually increased among sonographers in 1997. Studies on sonographers from the United States and Canada in 1997 reported an 84% incidence rate, which increased to 90% in 2008. Sonographers reported pain in the shoulders (76%), neck (74%), wrist (59%), back (58%) and hands (55%). Other studies on healthcare workers showed similar results. The most common location of MSDs were the shoulder (39%), back (38.1%), neck (37.5%) and wrist (29.4%) (7). The Rapid Entire Body Assessment (REBA) scoring system, established by Hignett and McAtamney in 2000, is a useful tool for evaluating the whole-body postural risk of MSDs in various tasks (6, 11).

Several academic literatures have reported on the ergonomic aspects of performing procedures that call for a display monitor, including bed and monitor height, the motor axis of the instrument, manipulating small devices and the force of contact between the ultrasonography probe and the patient’s body. Baker and Coffin (10) reported that ergonomic workstations decreased the frequency of WMSDs and boosted sonographer productivity. A proper ergonomic workspace must be learned and practiced by the operator performing the ultrasonographic technique. Sufficient time should be allotted to properly set up the ultrasonography workstation and operate it safely (10). However, in an emergency setting, this procedure can be constrained by the amount of time available, clinical setting and personnel shortages in a crowded ED (12).

A previous ergonomic study on interventional radiologists by Shinohara (13) reported that neuromuscular pain due to improper equipment positioning, which results in bad operator posture, is one of the most frequent ergonomic issues. Incorrect placement of the equipment can lead to unnecessary twisting movements, impaired manoeuvrability and pressure on the wrists, neck and back of the operator. Dexterity fatigue is linked to the manipulation of tiny calibre catheter placement during intravenous cannulation (13).

The operating surface height may also affect procedure ergonomics. On the basis of elbow variables, van Veelen et al. (14) determined that among six options, 0.7–0.8 elbow heights were the best operating surface height for laparoscopic surgery. Lower heights promoted back pain, whereas higher surfaces led to wrist, shoulder and neck pain (14).

Ergonomics aims to increase procedure effectiveness and efficiency along with comfort and safety. van Det et al. (15) reviewed the literature on randomised controlled and clinical trials and concluded that the short operating time of laparoscopic surgery due to positioning of the equipment during the process is a key factor in determining its effectiveness and efficiency. To minimise head and neck movement, the display monitor is positioned in front of the surgeon in line with the forearm-instrument motor axis (15). There is further evidence that highlights the reduced task time performance of ultrasonography-guided nerve block when the needle is inserted along the visual axis as opposed to across it (5). This step increased the operator’s comfort and safety while maintaining a 15° downward gaze and an ideal placement range of 50 cm–100 cm from the monitor (15).

The ideal ultrasonography probe orientation for venous cannulation is a topic of debate. The ultrasonography probe’s transverse orientation (TO) and longitudinal orientation (LO) are the two most frequently used methods for venous cannulation (16). Every approach has benefits and drawbacks depending on the vascular access.

To date, we have not experienced any ergonomics assessment of ultrasonography-guided procedures, including IJV cannulation, in the ED. We may determine which position has the MSD risk by evaluating the operator’s posture using the REBA score. Subsequently, preventative measures may be implemented for preventing WMSDs.

## Methods

A longitudinal study on simulated manikin was conducted in Hospital Universiti Sains Malaysia, a teaching hospital in Kubang Kerian, Kelantan. This study was approved by the Human Ethics Committee of Hospital Universiti Sains Malaysia, Kelantan. Sixty emergency medicine residents who provided their consent to participate were included in this study. They must have completed ultrasonography training, which involves ultrasonography-guided procedures such as central vein cannulation, and have at least 1 year of experience working in an ED.

The following were the exclusion criteria: participants who were pregnant; had a history of established musculoskeletal conditions, including prolapse intervertebral disc; had fracture records in the long bones or spine; had congenital abnormalities of the trunk and extremities; or had previous joint surgery. The Department of Emergency Medicine’s simulation room served as the study’s location. Every participant was enrolled when they were not performing their assigned duties.

To reduce the impact of confounding variables, a within-subject study design was used. Each participant was tasked with performing IJV cannulation under ultrasonographic guidance at two different table heights. The table height was customised according to the individual’s height by applying 0.7 and 0.5 elbow factors. Elbow height was defined as the distance measured from the participant’s olecranon tip (elbow point) when standing with the elbow bent at a 90° angle. Before the process, the height of each participant’s elbow was measured and the table height was adjusted on the basis of the 0.7 elbow factor (70% of the elbow height) and 0.5 elbow factor (50% of the elbow height) of their own height. For example, participant A, with a 110 cm elbow height, must perform cannulation at a 77 cm bed height using a 0.7 elbow factor and a 55 cm bed height using a 0.5 elbow factor.

At each table height, participants should perform two cannulations using each technique: TO and LO or also known as out-of-plane and in-plane technique, respectively. Therefore, each participant should complete four cannulations. Each cannulation procedure should take approximately 5 min–10 min. To preserve puncture accuracy, at least 5 min of rest were allowed to the participants between cannulations. The total duration of this study should take approximately 1 h.

### Data Collection

**Figure 1:**
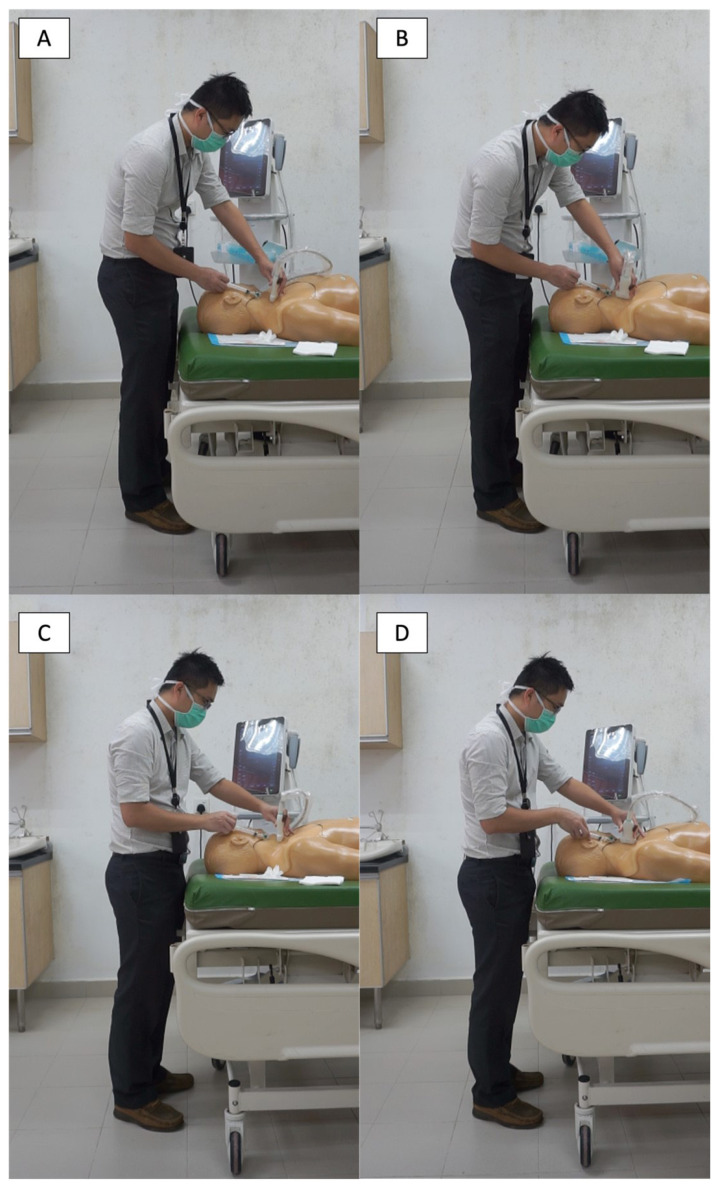
Four cannulation positions. A. table height of 0.5 elbow factor with transverse orientation (TO); B. table height of 0.5 elbow factor with longitudinal orientation (LO); C. table height of 0.7 elbow factor with TO and D. table height of 0.7 elbow factor with LO

Each participant should perform all four different positions of cannulation in a distinct sequential order, as shown in Figure 1. For example, first, the participants should perform the cannulation at a table height of 0.5 elbow factor (50% of the participant’s elbow height) with TO; in the second position, a table height of 0.5 elbow factor with LO was used; in the third position, a table height of 0.7 elbow factor (70% of the participant’s elbow height) with TO was used; lastly, a table height of 0.7 elbow factor with LO was employed.

The distinct sequences for each participant were developed from a two-stage sealed envelope method. The initial step involved determining the table height (A, 0.5; B, 0.7), followed by selecting the probe orientation (1, TO; 2, LO) in the subsequent stage. The potential produced sequences could include A1-A2-B1-B2, A2-A1-B1-B2, A1-A2-B2-B1, A2-A1-B2-B1 and so on. To minimise the influence of fatigue or the impact of one position on the performance of the next position, participants were not provided with a consistent sequence of positions.

### Research Tool

**Figure 2:**
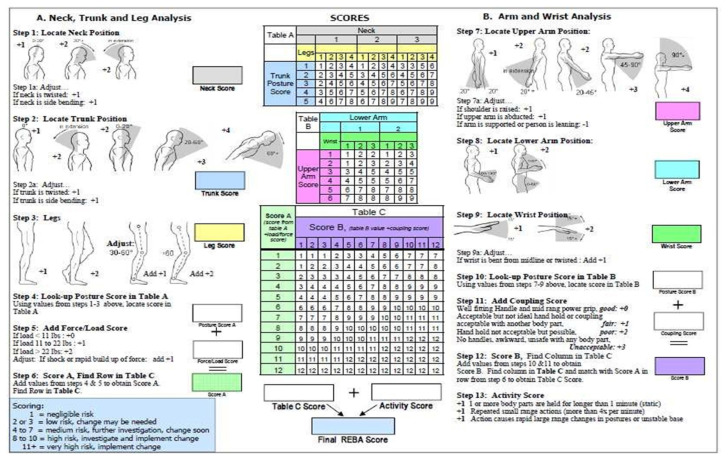
REBA worksheet

For each cannulation, the risk of MSD was assessed using the REBA assessment worksheet (Figure 2) (17). Each main body part was evaluated and scored. There were three primary portions of analysis referred to as scores A, B and C. Score A pertained to the study of the neck, trunk and legs, whereas score B was for analysing the upper arm, lower arm and wrist. Each part included a posture score scale and adjustment remarks that incorporated the elements of load/force involved in the activity and coupling factors of both arms. The score for each section can be obtained from the tables provided in the REBA worksheet. Score A was calculated by adding the table A score to the load/force score. Score B was calculated by adding the table B score and the coupling score for each hand. Score C was calculated on the basis of table C using the results of scores A and B. The REBA score was calculated by adding score C and the activity score. The REBA score represents MSD risk from 1 to 15. The ratings indicate MSD risk as negligible (score 1), low (score 2–3), medium (score 4–7), high (score 8–10) and very high (score 11+).

### Statistical Analysis

Data were analysed using Statistical Package for the Social Sciences (version 27.0, IBM, Armonk, NY, USA). Graphical presentation was created by Rstudio. Data distribution for normality checking was performed using the Shapiro-Wilk test. Median differences of the REBA score and *P*-value were determined using the Friedman test.

## Results

The demographic information of the participants is presented in Table 1. The study included 60 participants, with an equal distribution of 30 males (50%) and 30 females (50%). The age of the participants varied between 31 years old and 38 years old, with an average age of 33.7 years old with a standard deviation (SD) of 1.87. The average weight and height of the participants were 69.6 kg (SD = 14.91) and 1.64 m (SD = 0.08), respectively.

The median REBA scores at various probe and table heights are displayed in Table 2. The table height of 0.5 elbow factor with varied probe resulted in a median REBA score of 5.0 (interquartile range [IQR] = 0), whereas the table height of 0.7 elbow factor with varied probe had a median REBA score of 4.0 (IQR = 1). Based on the results of the Friedman test, we rejected the null hypothesis and observed a significant variation in REBA scores with varied probe and table heights (*P* < 0.001).

Five postures were regarded as ‘high risk’ according to the REBA method, with a score of 9. All these measurements were taken at a table height of 0.5 elbow factor, with three participants scored at TO probe and another two participants with LO probe (Table 2). The table height of 0.5 elbow factor scored higher as it was contributed from score A (analysis of the neck, trunk and legs), which was mainly influenced by trunk flexion (Table 3) (median score, 2.0 [IQR = 1]). Minimal variation was observed in the other component of score A, and the overall body part analysis of score B across all positions in table heights and probe orientations was similar.

The two best positions achieved by the REBA score of 2 were both rated as ‘low risk’. These positions were recorded at the table height of 0.7 elbow factor and each at a different probe orientation (Table 2).

## Discussion

Considering the increasing complaints of MSDs among medical staff, identifying the modifiable and therapeutic factors contributing to their MSD symptoms is crucial. Early intervention can help avoid and reduce operator fatigue and discomfort, thereby leading to a better work environment and enhanced performance. Several factors contribute to the ergonomics of ultrasonography-guided IJV cannulation. Besides the abovementioned findings, the height of the operating table and the manner the ultrasonography probe is held are among the factors contributing to MSD development.

Our findings showed that the table height of 0.7 elbow factor with varied probe orientations had a better score than the table height of 0.5 elbow factor of any probe orientation. This finding is consistent with those of previous literature on the ideal operating surface height for laparoscopic surgery (14). Although various table heights yielded significantly different results, both table heights with diverse probe orientations presented an equal risk of MSD development. According to the REBA score, all four positions exhibited a moderate level of risk, indicating the need for additional investigations and the potential execution of corrective measures (11, 17).

When the table height was set at a 0.5 elbow factor, we observed that the score increased. This was derived from score A (analysis of the neck, trunk and legs), which was mainly influenced by the trunk posture at a flexion of 0°–20°. Compared with the table height of 0.7 elbow factor, most of the participants performing the procedure were in the neutral trunk position (0°). Participants who scored the lowest REBA of 2, which was rated as ‘low risk’, had minimal bending of the torso, upper and lower limb joints while completing the task.

In general, as the operating surface is high, the operator should abduct the shoulder more, and flex the elbow and wrist. However, the study shows minimal variation in arm and wrist analysis across all positions, including varying table heights and probe orientations. The position of the neck scored nearly the same in all configurations owing to the fixed placement of the screen monitor on the left side of the bed, which maintains the in-line axis of the head to the monitor.

The preference of ultrasonography probe orientation for venous cannulation is debated. The most common ultrasonography probe orientations for venous cannulation are TO and LO. Each approach has advantages and disadvantages, depending on the vascular access. In 2018, a meta-analysis by Liu et al. (18) assessed TO and LO ultrasonography probe cannulation at various vascular locations for efficacy and safety. These two approaches had identical total and first-attempt IJV cannulation success rates according to trial sequential analysis (18). Ergonomically, REBA scoring shows no significant difference between LO and TO probe orientations. The neck, trunk, shoulders and legs are generally the same in both cases; however, the lower arm and wrist flexion slightly differs.

The study’s scope was limited owing to the small sample size, which solely comprised emergency medicine residents from a single centre. The use of a manikin may deviate from the characteristics of an actual human body.

## Conclusion

This study determined that a table height with a 0.7 elbow factor offers a superior position than a table height with a 0.5 elbow factor, despite both postures being classified as medium risk according to the REBA assessment. There is no discernible distinction between the probe orientation of TO and LO when evaluated at the same table height. Despite being a single-centre study with a limited sample size, it successfully addressed the necessity for additional assessment in enhancing emergency medical treatment from an ergonomic perspective, ultimately aiming to deliver exceptional care to the community.

## Figures and Tables

**Table 1:** Socio demographic characteristic of study sample

Variables	*n* (%)	Mean (SD)
Mean age (years old)		33.7 (1.87)
Gender		
Male	30 (50)	
Female	30 (50)	
Mean weight (kg)		69.6 (14.91)
Mean height (m)		1.64 (0.08)
BMI (kg/m^2^)		25.98 (5.16)
Years of service		
< 2 years	0 (0)	
2–5 years	14 (23.3)	
> 5 years	46 (76.67)	

Notes: SD = standard deviation; BMI = body mass index

**Table 2:** REBA score at across type of probe orientation and table height

Variable	REBA Score			*P*-value^a^

	Lowest, *n* (%)	Highest, *n* (%)	Median (IQR)	
TO probe at 0.5 elbow factor table height			5.0 (0)	< 0.001
LO probe at 0.5 elbow factor table height	4, 8 (13.3)	9, 3 (5.0)	5.0 (0)	
TO probe at 0.7 elbow factor table height	2, 1 (1.7)	7, 2 (3.3)	4.0 (1)	
LO probe at 0.7 elbow factor table height	2, 1 (1.7)	6, 1 (1.7)	4.0 (1)	

Notes: IQR = interquartile range;

a Related-Samples Friedman’s Two-Way Analysis of Variance;

TO = transverse orientation; LO = longitudinal orientation

**Table 2:** REBA score at across type of probe orientation and table height

Position	REBA score median (IQR)								

	Neck	Trunk	Legs	Upper arm	Lower arm	Wrist	Score A	Score B	Total score
LO probe at 0.5 elbow factor table height	3.0 (0)	2.0 (1)	1.0 (0)	2.0 (1)	1.0 (1)	2.0 (0)	4.0 (1)	2.0 (1)	5.0 (0)
TO probe at 0.5 elbow factor table height	3.0 (0)	2.0 (1)	1.0 (0)	2.0 (1)	1.0 (1)	2.0 (0)	4.0 (1)	2.0 (1)	5.0 (0)
TO probe at 0.7 elbow factor table height	3.0 (0)	1.0 (1)	1.0 (0)	2.0 (0)	1.0 (0)	2.0 (0)	3.0 (1)	2.0 (1)	4.0 (1)
LO probe at 0.7 elbow factor table height	3.0 (0)	1.0 (1)	1.0 (0)	2.0 (0)	1.0 (0)	2.0 (0)	3.0 (1)	2.0 (1)	4.0 (1)

Notes: IQR = interquartile range;

a Related-Samples Friedman’s Two-Way Analysis of Variance;

TO = transverse orientation; LO = longitudinal orientation
